# Our Journal

**Published:** 1858-02

**Authors:** 


### EDITORIAL AND MISCELLANEOUS.

### Our Journal.

We are making arrangements which we hope will result in a decided improvement to this Journal, and desire to express the determination to add to its interest continually, as our friends may enable us so to do; and to this end we would urge those who are now subscribers and contributors to exert themselves to extend its circulation, and increase the number and ability of its collaborators. It is true that we have now quite a large subscription list, but not half what we propose, or that would be necessary to justify us in furnishing the Journal of which we would desire to be the Editors.

